# Vascular endothelial growth factor expression is independent of hypoxia in human malignant glioma spheroids and tumours

**DOI:** 10.1054/bjoc.1999.0975

**Published:** 2000-01-18

**Authors:** M B Parliament, M J Allalunis-Turner, A J Franko, P L Olive, R Mandyam, C Santos, B Wolokoff

**Affiliations:** Divisions of Radiation and Experimental Oncology, Cross Cancer Institute, Edmonton, Alberta, Canada; British Columbia Cancer Research Center, Vancouver, British, Columbia, Canada

**Keywords:** hypoxia, glioma, vascular endothelial growth factor, misonidazole, flt-1

## Abstract

We recently showed that severe hypoxia was not universally present adjacent to necrosis in human glioma xenografts and spheroids established from the M059K, M006, M006X, M006XLo and M010b cell lines. Using these glioma models, we wished to test whether oxygen serves as a regulator of cellular VEGF expression in situ. In situ hybridization (ISH) and immunohistochemistry (IHC) were used to detect vascular endothelial growth factor (VEGF) mRNA and protein expression in sections of glioma xenografts and spheroids in which hypoxic regions and regions with well-oxygenated necrosis were identified on contiguous sections by use of the hypoxia-specific marker,^3^H-misonidazole. Independent validation of the presence of radiobiologically hypoxic cells in M006 xenografts was undertaken using the comet assay. Northern blotting analyses of monolayer cells demonstrated significant up-regulation of VEGF mRNA in the M006X line at oxygen concentrations of 6% and below. ISH analysis of VEGF mRNA showed unexpectedly strong staining for VEGF mRNA across the entire viable rim of M006X and M006XLo glioma spheroids. Similarly, in virtually all xenograft tumours of the M059K, M006 and M010b lines, VEGF ISH showed similar staining across all regions of healthy cells up to the border of necrosis. Only in one M006X tumour was there a suggestion of increased VEGF expression in cells adjacent to necrosis. IHC for VEGF showed good concordance with the ISH results. IHC analysis of the VEGF receptor flt-1 showed strong tumour cell staining in M006XLo glioma cells. In human glioma spheroids and xenograft tumours, regions of severe hypoxia do not correspond to areas of up-regulated VEGF expression; in fact, VEGF expression is quite uniform. Furthermore, this and our previous study demonstrate that levels of VEGF expression vary among sublines (M006, M006X and M006XLo) derived from a single human glioma specimen. © 2000 Cancer Research Campaign


*British Journal of Cancer* (2000) **82**(3), 635–641
© 2000 Cancer Research Campaign
DOI: 10.1054/ bjoc.1999.0975, available online at http://www.idealibrary.com on IDEAL®

### Vascular endothelial growth factor expression is independent of hypoxia in human malignant glioma spheroids and tumours

**MB Parliament[1], MJ Allalunis-Turner[2], AJ Franko[2], PL Olive[3], R Mandyam[2], C Santos[2] and B Wolokoff[2]**

Divisions of [1]Radiation and [2]Experimental Oncology, Cross Cancer Institute, Edmonton, Alberta, Canada; [3]British Columbia Cancer Research Center, Vancouver, British Columbia, Canada

**Summary** We recently showed that severe hypoxia was not universally present adjacent to necrosis in human glioma xenografts and spheroids established from the M059K, M006, M006X, M006XLo and M010b cell lines. Using these glioma models, we wished to test whether oxygen serves as a regulator of cellular VEGF expression in situ. In situ hybridization (ISH) and immunohistochemistry (IHC) were used to detect vascular endothelial growth factor (VEGF) mRNA and protein expression in sections of glioma xenografts and spheroids in which hypoxic regions and regions with well-oxygenated necrosis were identified on contiguous sections by use of the hypoxia-specific marker, $^{3}$H-misonidazole. Independent validation of the presence of radiobiologically hypoxic cells in M006 xenografts was undertaken using the comet assay. Northern blotting analyses of monolayer cells demonstrated significant up-regulation of VEGF mRNA in the M006X line at oxygen concentrations of 6% and below. ISH analysis of VEGF mRNA showed unexpectedly strong staining for VEGF mRNA across the entire viable rim of M006X and M006XLo glioma spheroids. Similarly, in virtually all xenograft tumours of the M059K, M006 and M010b lines, VEGF ISH showed similar staining across all regions of healthy cells up to the border of necrosis. Only in one M006X tumour was there a suggestion of increased VEGF expression in cells adjacent to necrosis. IHC for VEGF showed good concordance with the ISH results. IHC analysis of the VEGF receptor flt-1 showed strong tumour cell staining in M006XLo glioma cells. In human glioma spheroids and xenograft tumours, regions of severe hypoxia do not correspond to areas of up-regulated VEGF expression; in fact, VEGF expression is quite uniform. Furthermore, this and our previous study demonstrate that levels of VEGF expression vary among sublines (M006, M006X and M006XLo) derived from a single human glioma specimen. © 2000 Cancer Research Campaign

**Key words**: hypoxia; glioma; vascular endothelial growth factor; misonidazole; flt-1

Among the various adaptive responses to cell and tissue hypoxia is that of neoangiogenesis. This process occurs under physiological and pathophysiological conditions. It is becoming apparent that primary brain tumours of astrocytic lineage, especially glioblastoma multiforme, exhibit hypoxia-induced neoangiogenesis in vivo through induction of the endothelial cell-specific mitogen, vascular endothelial growth factor (VEGF) (Shweiki et al, 1992; Plate et al, 1992, 1994). A strong correlation was found between intense VEGF expression and severe hypoxia in situ in HT29 and EMT-6 spheroids (Waleh et al, 1995).

Recently we have observed variable presence of hypoxia adjacent to necrosis in some xenografted glioma lines and spheroids (Parliament et al, 1997; Franko et al, 1998). The results were interpreted in terms of regional variations in oxygen consumption, based on the concept of modulation of oxygen consumption in regions of oxygen and nutrient deprivation in solid tumours (Hochachka et al, 1996). We were interested in determining the pattern of VEGF expression in situ, particularly in the nutrient-deprived regions of viable cells adjoining necrosis, which may or may not be severely hypoxic in human glioma spheroids and xenografts. We recently showed that the M006XLo line has elevated expression of VEGF under aerobic conditions, with modest hypoxic induction compared to the M006X line (Allalunis-Turner et al, 1999). In this report we have examined the oxygen dependence of VEGF mRNA expression in the M006X line undergoing mild hypoxic stress (6% oxygen ($O_2$)), and we have also examined VEGF expression in spheroids of both M006X and M006XLo lines and in xenograft tumours of the M059K, M006 and M010b lines. We also sought to independently validate the presence of radiobiologically hypoxic cells in situ in M006 tumours using the comet assay of in-situ DNA damage (Olive et al, 1997, 1998).

Finally, we have performed preliminary experiments to assess the presence of tumour-cell VEGF receptor (Vaisman et al, 1990) expression which has previously been seen in melanoma and ovarian carcinoma lines (Boocock et al, 1995; Liu et al, 1995), suggesting autocrine/paracrine growth stimulation.

*Received 10 February 1999*
*Revised 20 July 1999*
*Accepted 23 July 1999*

*Correspondence to:* MB Parliament, 11560 University Avenue, Edmonton, Alberta, Canada, T6G 1Z2

### MATERIALS AND METHODS

### Cell lines, spheroids and xenografts

Details of the origin and characterization of the glioma cell lines used in this study have been previously published (Parliament et al, 1997; Allalunis-Turner et al, 1991). The M0059K (passage

153) and M006 (passage 113) lines were established from biopsies of grade 4 astrocytomas; the M010b (passage 44) line was derived from a grade 3 anaplastic astrocytoma. The M006X (passage 131) subline was derived from a xenografted M006 tumour growing in a severe combined immunodeficient (SCID) mouse and disaggregated by mechanical and enzymatic methods as described previously (Parliament et al, 1997). M059KX (passage 165) and M010bX (passage 54) sublines were similarly derived from xenografts originating from the M059K and M010b lines respectively. The M006XLo subline was established from M006 spheroids (passage 129) which had been exposed continuously to 0.6% oxygen for 13 days, beginning 1 week after initiation in air. The spheroids were disaggregated with 0.25% trypsin, and the cells were grown in monolayer culture for four passages (5 weeks), and injected subcutaneously (s.c.) in SCID mice. One tumour was disaggregated to yield a new line designated M006XLo, which was propagated in monolayer culture (Parliament et al, 1997). Details of the initiation and growth of spheroids were as described in Franko et al (1998). M006XLo, M006X, M059K and M010b tumours were raised s.c. in the flanks of NOD/SCID mice. Details of the initiation and growth of tumour xenografts were as described in Parliament et al (1997). All animal experiments were performed according to guidelines for the use of animals in research established by the Canadian Council for Animal Care.

### Northern blotting

Glioma cells were grown as monolayers under standard incubator conditions (37°C, 5% carbon dioxide–95% air). To study the effect of changes in the gas-phase $O_2$ concentration on VEGF expression, a gas exchange manifold system utilizing a series of leakproof aluminium chambers (Koch et al, 1979) was used to create atmospheres of varying proportions of oxygen in 5% carbon dioxide–95% $N_2$. Total RNA was isolated from cells cultured for 24 h under aerobic or hypoxic conditions using the guanidinium–silica gel column method (Qiagen). Twenty micrograms of total RNA were electrophoresed in each lane. After transfer, the blots were hybridized with $^{32}$P random-labelled probes to β-actin (gift from Dr Roseline Godbout) or VEGF (gift from Dr Harold Dvorak). The 204-bp human VEGF probe was originally prepared by reverse transcription polymerase chain reaction (RT-PCR) with oligonucleotide primers [(forward): 5′-CGCGGATCCAGGAGTACCCTGATGAG-3′; (reverse) 5′-CCGGAATTCACATTTGTGTGCTGT-3′] based on the human VEGF sequence (Berse et al, 1992). This probe recognizes all VEGF transcripts and does not discriminate among alternatively spliced transcripts (Berse et al, 1992). To quantify changes in VEGF mRNA expression, the autoradiograms which detected $^{32}$P decay were scanned using a PDQUEST densitometer (*pdi* Inc., Huntington Stn, NY, USA). Optical densities of the regions of the VEGF and β-actin bands were determined using *pdi* Quantity One® scanning and analysis software. VEGF mRNA optical densities were normalized using β-actin mRNA of each sample as a control. Increases in mRNA following exposure to hypoxic conditions were expressed as a proportion of the aerobic (18% $O_2$) culture expression levels.

### Comet assay

Unanaesthetized NOD/SCID mice bearing M006 tumours were used for experiments when the tumours reached a size of 300–500 mg. Mice were restrained in lucite jigs and breathed room air. The tumours were irradiated with 15 Gy 250 kV X-rays at a dose rate of 3.3 Gy min$^{-1}$. Tumours were excised within 30 s at the end of radiation exposure and disaggregated in ice-cold phosphate-buffered saline (PBS). Cell suspensions were mixed with agarose (final concentration 0.75%), lysed with an alkaline high salt lysis solution, rinsed in alkali, and exposed to 0.6 volts cm$^{-1}$ in an electrophoresis chamber as previously described (Olive et al, 1997). DNA was stained with propidium iodide and individual cell ‘comets’ were viewed using a Zeiss epifluorescence microscope attached to an intensified solid state charge device camera and image analysis system (Olive et al, 1997). The hypoxic fraction was estimated by iterative fitting of histograms of ‘tail moment’ (product of the percentage of DNA in the comet tail multiplied by the distance between the means of the head and tail distributions) with two normal distributions, representing the aerobic and hypoxic populations as described previously (Olive et al, 1997).

### Histologic preparation

After growth to diameters of 800–1000 μm spheroids of the M006 and M006XLo lines were labelled under aerobic conditions with 50 μM $^{3}$H-misonidazole for 3 h as described previously (Franko et al, 1998). The specific activity of $^{3}$H-misonidazole was $1.045 \times 10^{10}$ Bq mol$^{-1}$. Tumours were labelled with $^{3}$H-misonidazole with three injections (of the same specific activity drug as noted above) at 1-h intervals into mice at a dose equivalent to a whole body concentration of 50 μM, as described previously (Parliament et al, 1997). The spheroids and tumours were either fixed in 10% buffered formalin at 4°C overnight, then at room temperature for at least 3 days, or fixed in methanol–chloroform–glacial acetic acid (6:3:1 by volume; methanol-Carnoy’s) at 4°C for 30 min, then overnight at room temperature. The material was then embedded in wax, cut at 5 μm and mounted on poly-L-lysine-coated slides.

### VEGF ISH

The human VEGF 204 bp probe described above was labelled with biotin using a T7 RNA polymerase using biotin-14-CTP (Gibco-BRL) according to manufacturer’s instructions. Spheroid and xenograft (M006X) sections, which had been fixed with formalin, were dewaxed and blocked with levamisole to block detection of endogenous biotin. In situ hybridization (ISH) detection utilized streptavidin linked to NBT (Oligo Colour Kit, Amersham Life Sciences). Semi-quantitative assessment (after Plate et al, 1994) of blue colour reaction was undertaken and compared to $^{3}$H-misonidazole autoradiography on serial sections (see below). The blue colour intensity was graded by one observer as: (–) = absent; (+) = light; (++) = moderate; (+++) = heavy; and (++++) = very heavy.

### VEGF immunohistochemistry

Deparaffined slides underwent unmasking with hot citric acid (2.1 g L$^{-1}$ at pH 7.3 for 30 min at 70°C), then were rinsed with PBS containing 10% goat serum. Slides were then incubated with the primary rabbit anti-human VEGF antibody, 1:150 (Santa Cruz A-20 raised to amino acid residues 1–20, recognizing VEGF 121, 165, 189 splice variants). The primary antibody was detected using a Fast Red detection kit (BioGenex). Slides were lightly

*© 2000 Cancer Research Campaign*

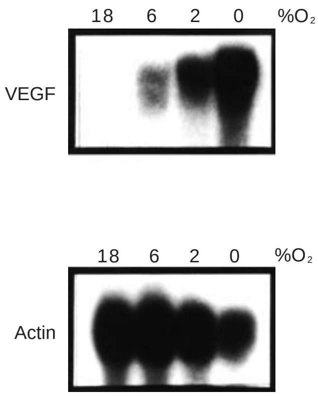

**Figure 1** Northern blot analysis of VEGF mRNA expression in M006X cells. Total RNA was isolated from cells incubated for 24 h under various oxygen concentrations as follows: lane 1, 18% $O_2$; lane 2, 6% $O_2$; lane 3, 2% $O_2$; lane 4, nitrogen counterstained with haematoxylin, in order to emphasize positive immunohistochemistry (IHC) staining. On control slides, the antibody was neutralized by incubation with a tenfold excess of control VEGF peptide (Santa Cruz P-20) prior to the anti-VEGF step. Additional controls in preliminary experiments used rabbit IgG in place of the primary antibody.

### $^3$H-misonidazole autoradiography

Sections adjacent to those used for ISH and IHC were dewaxed, dipped in NTB-2 emulsion and exposed for 4–6 weeks. After developing and fixing, the sections were stained through the emulsion with haematoxylin and eosin. Grain density was counted regionally at 1000× using a 10 × 10 μm microscope grid.

### VEGF receptor (flt-1) IHC

M006XLo slides were prepared as for VEGF IHC. These slides were then incubated with a polyclonal rabbit anti-human flt-1 antibody (Santa Cruz). The primary antibody was detected with the Fast Red detection kit (BioGenex).

### RESULTS

### Northern blotting

Northern blot analysis of VEGF mRNA expression in M006X cells incubated at various $O_2$ concentrations is shown in Figure 1. VEGF expression increased markedly with exposure to increasing degrees of hypoxia. Levels of β-actin mRNA were comparable at 18, 6 and 2% $O_2$, and were slightly decreased under anoxia. Normalizing the (VEGF/actin) ratio in room air to unity, the degree of VEGF induction observed under 6% $O_2$, 2% $O_2$, and anoxia (100% $N_2$) was as follows: 3.7-, 9.5-, and 29.8-fold respectively.

### Hypoxic fraction of M006 xenografts measured using the comet assay

In 12 tumours from air-breathing mice, the mean hypoxic fraction was 8.9 ± 7.0%, (range 0–21%). These results are summarized in Table 1.

**Table 1** Hypoxic fraction of M006 glioma xenograft tumours measured using the comet assay

| Tumour number | Treatment | Proportion of hypoxic cells (curve fitting) |
|---|---|---|
| 1 | Air-breathing | 0.10 |
| 2 | Air-breathing | 0.034 |
| 3 | Air-breathing | 0.01 |
| 4 | Air-breathing | 0 |
| 5 | Air-breathing | 0.137 |
| 6 | Air-breathing | 0.042 |
| 7 | Air-breathing | 0.071 |
| 8 | Air-breathing | 0.21 |
| 9 | Air-breathing | 0.107 |
| 10 | Air-breathing | 0.075 |
| 11 | Air-breathing | 0.067 |
| 12 | Air-breathing | 0.21 |
| 13 | Clamped | 0.81 |
| Mean ± SD | Air-breathing | 0.089 ± 0.07 |

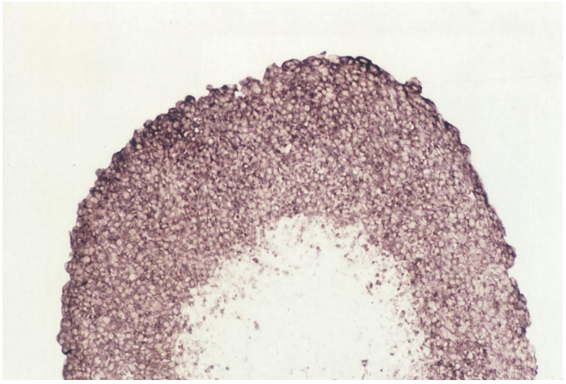

**Figure 2** VEGF ISH from M006XLo spheroid (10×)

### Relationship of VEGF mRNA expression to hypoxia marker binding in glioma spheroids and xenografts

Aerobic M006XLo spheroids (Figure 2) and M006X spheroids showed strong staining for the hybridized VEGF probe across the entire viable rim. Semiquantitative assessment of ISH staining was made and compared with the intensity of $^3$H-misonidazole labelling on contiguous 5-μm sections (Table 2). Heterogeneity of $^3$H-misonidazole labelling adjacent to necrosis was seen from spheroid to spheroid within the same experimental flask as reported previously (Franko et al, 1998). Some spheroids displayed heavy labelling in the inner five cell layers adjacent to necrosis, indicating a substantial gradient of [$O_2$], and severe hypoxia adjacent to necrosis, while others were more lightly labelled indicating aerobic levels of oxygen throughout the spheroids (Franko et al, 1998). There was no apparent relationship between VEGF expression and hypoxic marker binding, although in one of ten spheroids, elevated $^3$H-misonidazole binding and VEGF ISH staining were seen to co-localize to the innermost cells. A similar relationship between VEGF and hypoxia marker binding existed in M006X spheroids, which show lower levels of aerobic expression of VEGF in vitro than the M006XLo line (data not shown).

© 2000 Cancer Research Campaign

**Table 2** $^{3}$H-miso autoradiography vs VEGF ISH in M006XLo glioma spheroids

| Spheroid number | $^{3}$H-miso grain density[a] | | VEGF ISH intensity[b] | |
|---|---|---|---|---|
| | Outer layers | Inner layers | Outer layers | Inner layers |
| 1 | + | + | + | + |
| 2 | + | + | + | + |
| 3 | + | + | ++ | +++ |
| 6 | + | + | +++ | +++ |
| 10 | + | + | ++++ | +++ |
| 4 | + | ++ | ++ | ++ |
| 5 | + | ++ | +++ | ++ |
| 8 | + | +++ | + | ++ |
| 9 | + | +++ | +++ | ++++ |

[a]Each extra (+) indicates an increase in grain density by a factor of approximately 8. [b]Intensity was graded by one observer as (–) = absent; (+) = light; (++) = moderate; (+++) = heavy; (++++) = very heavy.

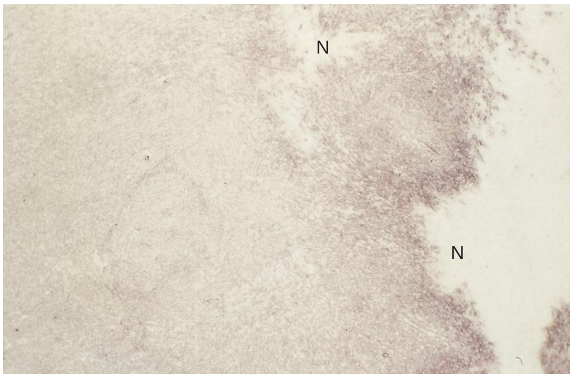

**Figure 3** VEGF ISH in M006X xenograft (4×). Regions of necrosis (N) are shown

VEGF ISH in M006X tumours showed uniform staining in all tumours examined except one, where there was increased staining adjacent to approximately 20% of necrotic regions (Figure 3A, 4×; Figure 3B 10×). Five tumours were assessed, with identical findings. Similar findings were also noted for M006XLo (two tumours), M010bX (one tumour) and M0059KX (two tumours).

### VEGF IHC

Immunoreactivity to the anti-VEGF antibody was superior for tumours and spheroids fixed with methanol-Carnoy's compared to those fixed with formalin. The staining was more intense and the concentration difference between non-specific staining with control IgG and acceptable staining with the antibody was at least threefold better for methanol-Carnoy's. Control sections in which the primary anti-VEGF antibody was neutralized by a large excess of control VEGF peptide were uniformly negative. In all tumours assessed of the M059KX, M006X, M006XLo and M010bX lines (four tumours from each), and in M006XLo spheroids, immunodetection of VEGF protein showed similar staining across all regions of healthy cells up to the border of necrosis (Figure 4A). In preliminary experiments which used a wide range of dilution of the primary antibody, the lightest staining visible appeared to be similarly uniform, with no suggestion of preferential staining of cells adjacent to necrosis. On contiguous sections, simultaneous assessment of hypoxia marker binding indicated marked heterogeneity, consistent with previous studies (Parliament et al, 1997). These tumour regions included cells exhibiting a full range of $^{3}$H-misonidazole grain densities (from fully aerobic to hypoxic). A similar result was apparent using formalin-fixed tissue (data not shown). These results indicate an absence of a correlation in situ between [$O_2$] and VEGF protein expression.

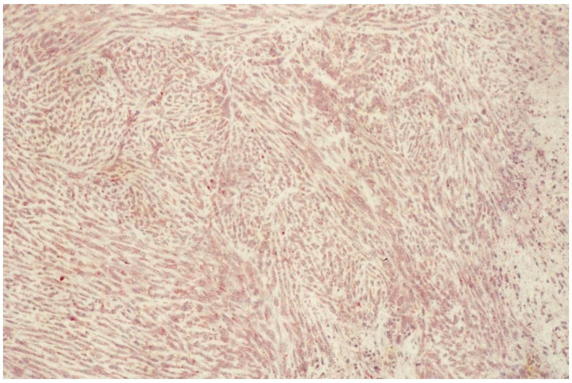

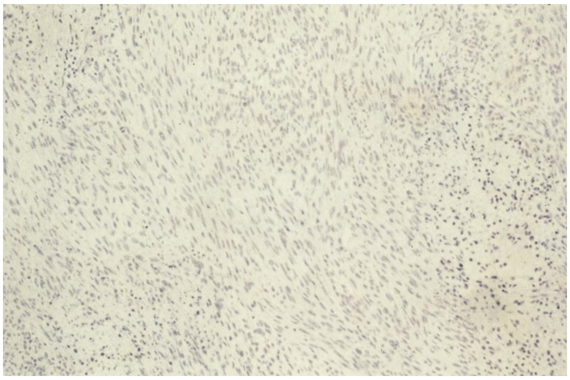

**Figure 4** (**A**) VEGF IHC in M006XLo xenograft (haematoxylin and eosin counterstain, 10×). (**B**) VEGF IHC control in M006XLo xenograft, neutralized with tenfold excess of control peptide (10×)

### VEGFR (flt-1) IHC

Using a similar protocol to detect VEGFR (flt-1) in M006XLo cells, we observed strong immunostaining for flt-1 in all tumour cells of this preparation (Figure 5).

 *© 2000 Cancer Research Campaign*

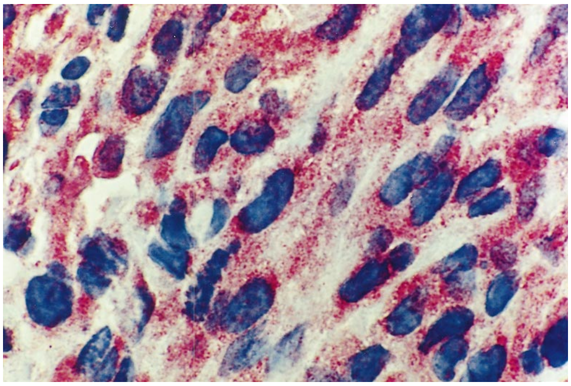

**Figure 5** VEGFR-1 (flt-1) IHC in M006XLo xenograft (haematoxylin and eosin counterstain, 100×)

### DISCUSSION

The understanding of neoangiogenesis in glioblastoma multiforme has advanced significantly in the last 4–5 years. Indeed, the presence of necrosis and vascular proliferation distinguishes glioblastoma multiforme from most other malignant brain tumours including astrocytomas (Germano et al, 1989). Previous investigators (Plate et al, 1992; Shweiki et al, 1992) noted marked up-regulation of VEGF expression in pallisading cells adjacent to necrosis and also in other small anaplastic cells at some distance from necrosis. It is now certain that in addition to hypoxic stress (Shweiki et al, 1992; Damert et al, 1997), VEGF may be induced by $CoCl_2$ (Goldberg and Schneider, 1994); nitric oxide (Chin et al, 1997); tumour necrosis factor-α (TNF-α) (Ryuto et al, 1996); glucose deprivation (Stein et al, 1995); platelet derived growth factor-BB; transforming growth factor-β (TGF-β) (Brogi et al, 1994; Frank et al, 1995); and interleukin-1β (Li et al, 1995). The presence of multiple metabolic and growth factor modulators of this endothelial mitogen imply a complex in vivo regulation. In contrast to the finding of co-localization of VEGF expression and hypoxia using the marker EF5 in HT29 and EMT-6 spheroids (Waleh et al, 1995), we found discordant results in several human glioma lines grown as multicellular spheroids and xenografts. Both VEGF ISH and IHC revealed virtually ubiquitous staining regardless of the proximity to nutrient capillaries or necrosis (Figures 3 and 4A, B). Only in a single xenograft tumour (M006X) was there a suggestion of increased VEGF mRNA expression adjacent to necrosis. Low power photomicrographs have been used to emphasize general uniformity of both VEGF ISH and IHC staining, with the ISH results confirmed by IHC.

Specific riboprobes that allow for the identification of the 121, 165, 189 and 206 amino acid isoforms of VEGF have been developed. The VEGF probe used in these studies, however, does not distinguish between the various VEGF mRNA species. In cardiac myocytes (Levy et al, 1995*a*), no significant changes in relative isoform amounts were observed in response to hypoxic stress. Given that the induction of VEGF mRNA species, using the probe in our Northern analysis, results in the classically described picture of VEGF mRNA induction with hypoxia, it is unlikely that the absence of variations in expression of VEGF in situ is simply related to changes in the proportions of isoforms without a change in total transcript number.

The findings in the current models differ from those originally described (Shweiki et al, 1992) in patient-derived primary biopsies. Every effort has been made to use glioma cell lines which are preserved in an early passage stage after explantation (Parliament et al, 1997). However, in situ oxygenation was not assessed in the original study (Shweiki et al, 1992) of VEGF overexpression; hypoxia was merely presumed to exist adjacent to necrosis. To our knowledge, our data represent the first examination of the relationship between VEGF expression and hypoxia in a solid glioma tumour model to date. A similar correlative study would be possible in patients, but would require in situ hypoxia marker labelling prior to biopsy (Urtasun et al, 1986). The hypoxic fraction values obtained with the M006 xenografts for the comet assay are close to those of the $C_3H$ murine mammary tumour under air-breathing conditions (Olive et al, 1997) and are consistent with our $^3$H-misonidazole data suggesting that most but not all M006 xenografts contain substantial radiobiological hypoxia.

The oxygen dependence of misonidazole binding is slightly different from the hypoxic marker EF5 (Koch et al, 1995). EF5 shows less inhibition of binding at moderate oxygen tensions, whereas misonidazole binding is inhibited by oxygen in a somewhat more complex fashion down to levels of hypoxia which would be considered significant from the point of view of induction of radiation resistance (Chapman, 1984). In spheroids we have also seen heterogeneous binding patterns of pimonidazole (Kennedy et al, 1997), adjacent to the necrotic centres in a fashion identical to that seen with misonidazole (AJ Franko, unpublished data). Therefore, the findings in this study do not appear to be an artifact of one particular hypoxia marker. Lack of correlation between hypoxia marker binding and VEGF expression in xenograft tumours is somewhat complex in that intratumoural drug distribution may be suboptimal because of the aberrant tumoural vasculature. However, glioma spheroids (Franko et al, 1998), in which nitroimidazole drug penetration is not impaired, also reveal substantial variation in hypoxia marker binding in the viable cells adjacent to necrosis, a pattern similar to that seen in xenograft tumours.

From the Northern analysis it is apparent that severe, pathophysiological levels of hypoxia are not necessary for the up-regulation of VEGF messenger RNA and protein expression; indeed substantial upregulation is evident at modest levels of $O_2$, which would be similar to the physiological hypoxic conditions noted in many mammalian organ systems. Our results are in agreement with those of Leith and Michelson (1995) who showed that modest hypoxia (i.e. 10% $O_2$) is also sufficient to cause some induction of VEGF expression in the clone A human colonic adenocarcinoma cell line. VEGF production is known to be mediated by both transcriptional and post-transcriptional mechanisms (Levy et al, 1995*b*, 1997). It is interesting to note that transcriptional regulation of VEGF expression, through binding of the hypoxia-inducible factor-1 (HIF-1) to a 28 bp site in the VEGF 5′ promoter (Wang et al, 1995; Jiang et al, 1996) also occurs at physiological levels of hypoxia with half maximal response between 1.5 and 2% $O_2$ (Jiang et al, 1996). Post-transcriptional regulation of VEGF mRNA levels appears to be due to reversible stabilization of VEGF mRNA by hypoxia (Shima et al, 1995; Stein et al, 1995; White et al, 1995; Levy et al, 1996). However, constitutive stabilization of VEGF can occur through inactivation of the von Hippel–Lindau tumour suppressor gene (Wizigmann-Voos et al, 1995; Gnarra et al, 1996; Iliopoulos et al, 1996; Levy et al, 1996). We are currently investigating the possibility that such post-transcriptional stabilization is involved in the constitutive expression of VEGF by the lines M006XLo and M059K.

© 2000 Cancer Research Campaign

The Northern blot data for M006X cells in vitro demonstrate that VEGF expression is unregulated by 3.5-fold following a reduction in $pO_2$ from 18% to the more physiologically relevant level of 6%. Furthermore, we have recently demonstrated IHC detection of increased expression of VEGF protein in cultured hypoxic (0.6 and 2% $O_2$) M006X cells (Allalunis-Turner et al, 1999). In vitro, the percentage of M006X cell staining positively by IHC for VEGF increased from 17% under fully oxygenated conditions to 97% (under 2% $O_2$). These data indicate that much of the VEGF mRNA induction detected by Northern blotting is a result of increase in the proportion of cells expressing VEGF rather than a uniform increase in VEGF mRNA in most cells. A change in staining pattern of this type would have been clearly visible in the IHC of the spheroids and tumours. These data are also compatible with the detection of VEGF mRNA and protein in presumably well oxygenated tumour cells adjacent to blood vessels in vivo. However, the Northern data do not provide an explanation for the lack of an increase in VEGF expression with increasing distance from blood vessels, and particularly the absence in almost all tumours of elevated expression in severely hypoxic cells adjacent to necrosis. An increase in expression by at least fourfold (possibly eightfold) in the latter areas was predicted by the Northern data, and clearly this was not observed. The possible biological reasons for the disparity between our in vitro and in vivo results require further investigation.

Up to 50% of the necrotic areas in M006 and M006XLo spheroids and M059K and M006 xenografts were reported to be well oxygenated (Parliament et al, 1997; Franko et al, 1998), and those observations are confirmed by the data reported here. The cause of cell death in these regions has not been identified, although we have postulated that it is related to glucose insufficiency (Franko et al, 1998), which is known to up-regulate VEGF expression (Stein et al, 1995). The absence of up-regulation of VEGF expression in cells adjacent to both aerobic and hypoxic necrosis suggests that its regulation in glioma cells is remarkably resistant to various types of microenvironmental stress.

Recently, a quantitative immunohistochemical comparison of hypoxia and VEGF protein expression in squamous cell carcinomas of the uterine cervix and head and neck has been reported (Raleigh et al, 1998). In this study in which patients received the hypoxia marker pimonidazole prior to biopsy, no correlation was seen between hypoxia and VEGF protein expression on quantitative image analysis of histologic sections. The authors considered the possibility that the lack of a correlation was a consequence of the fact that the half-maximal inhibition of 2-nitroimidazole binding by oxygen (0.4% gas-phase concentration) occurs at a lower $[O_2]$ than half-maximal VEGF up-regulation (0.8–2.2% gas-phase concentration) in mammalian cells. While this means that pimonidazole labelling would not identify all cells capable of inducing VEGF expression via a hypoxia-driven mechanism, the authors argued that substantial gradients of VEGF expression should have been observed in the cells separating blood vessels and severely hypoxic cells. These were not observed, which lends support to our observation that VEGF expression is independent of hypoxia in these glioma models.

While one may speculate that cell lines overexpressing VEGF when grown as tumours in vivo may achieve a growth advantage because of a 'pro-angiogenic' phenotype, we have considered another possible hypothesis. Reports of expression of VEGF receptors KDR (Boocock et al, 1995; Liu et al, 1995) and flt-1 (Boocock et al, 1995) have suggested a possible autocrine/paracrine growth stimulation by VEGF in certain melanoma and ovarian carcinoma lines. In view of this possibility, VEGFR-1 (flt-1) expression in the M006XLo xenograft was analysed. Strong, widespread expression of VEGFR-1 was detected in tumour cells. Previous studies utilizing biopsies from patients with gliomas have reported only tumour endothelial cell expression of VEGF receptors (Plate et al, 1994). Confirmatory studies are required to establish whether autocrine stimulation of growth is occurring in these lines.

### ACKNOWLEDGEMENTS

This study was supported by an award from the National Cancer Institute of Canada, with funds provided by the Canadian Cancer Society (to MBP), and also by NIH grant #CA-37879 (to PLO).

### REFERENCES

Allalunis-Turner MJ, Day III RS, McKean JDS, Petruk KC, Allen PB, Aronyk KE, Weir BK, Huyser-Wierenga D, Fulton DS and Urtasun RC (1991) Glutathione levels and chemosensitizing effects of buthionine sulfoximine in human malignant glioma cells. *J Neuro-Oncol* **11:** 157–164

Allalunis-Turner MJ, Franko AJ and Parliament MB (1999) Modulation of oxygen consumption rate and VEGF mRNA expression in human malignant glioma cells by hypoxia. *Br J Cancer* **80:** 104–109

Berse B, Brown LF, Van De Water L, Dvorak HF and Senger DR (1992) Vascular permeability factor (vascular endothelial growth factor) gene is expressed differentially in normal tissues, macrophages, and tumors. *Mol Biol Cell* **3:** 211–220

Boocock CA, Charnock-Jones DS, Sharkey AM, McLaren J, Barker PJ, Wright KA, Twentyman P and Smith SK (1995) Expression of vascular endothelial growth factor and its receptors flt and KDR in ovarian carcinoma. *J Natl Cancer Inst* **87:** 506–516

Brogi E, Wu T, Namiki A, and Isner JM (1994) Indirect angiogenic cytokines upregulate VEGF and bFGF gene expression in vascular smooth muscle cells, whereas hypoxia upregulates VEGF expression only. *Circulation* **90:** 649–652

Chapman JD (1984) The cellular basis of radiotherapeutic response. *Radiat Phys Chem* **24:** 283–291

Chin K, Kurashima Y, Ogura T, Tajiri H, Yoshida S, Esumi H (1997) Induction of vascular endothelial growth factor by nitric oxide in human glioblastoma and hepatocellular carcinoma cells. *Oncogene* **15**: 437–442

Damert A, Machin M, Breier G, Fujita MQ, Hanahan D, Risau W and Plate KH (1997) Up-regulation of vascular endothelial growth factor expression in a rat glioma is conferred by two distinct hypoxia-driven mechanisms. *Cancer Res* **57**: 3860–3864

Frank S, Hubner G, Breier G, Longaker MT, Greenhalgh DG and Werner S (1995) Regulation of vascular endothelial growth factor expression in cultured keratinocytes. Implications for normal and impaired wound healing. *J Biol Chem* **270**: 12607–12613

Franko AJ, Parliament MB, Allalunis-Turner MJ and Wolokoff BG (1998) Variable presence of hypoxia in M006 human glioma spheroids and in spheroids and xenografts of clonally derived sublines. *Br J Cancer* **78**: 1261–1268

Germano IM, Ito M, Cho KG, Hoshino T, Davis RL and Wilson CB (1989) Correlation of histopathological features and proliferative potential of gliomas. *J Neurosurg* **70**: 701–706

Gnarra JR, Zhou S, Merrill MJ, Wagner R, Krumm A, Papavassiliou E, Oldfield EH, Klausner RD and Linehan WM (1996) Post-transcriptional regulation of vascular endothelial growth factor mRNA by the product of the VHL tumor suppressor gene. *Proc Natl Acad Sci USA* **93**: 10589–10594

Goldberg MA and Schneider TJ (1994) Similarities between the oxygen-sensing mechanisms regulating the expression of vascular endothelial growth factor and erythropoietin. *J Biol Chem* **269**: 4355–4359

Hochachka PW, Buck LT, Dahl CJ and Land SC (1996) Unifying theory of hypoxia tolerance: molecular/metabolic defense and rescue mechanisms for surviving oxygen lack. *Proc Natl Acad Sci USA* **93**: 9493–9498

Iliopoulos O, Levy AP, Jiang C, Kaelin WG Jr and Goldberg MA (1996) Negative regulation of hypoxia-inducible genes by the von Hippel–Lindau protein. *Proc Natl Acad Sci USA* **93**: 10595–10599

Jiang B-H, Semenza GL, Bauer C and Marti HH (1996) Hypoxia-inducible factor 1 levels vary exponentially over a physiologically relevant range of $O_2$ tension. *Am J Physiol* **271(**Cell Physiol**. 40)**: C1172–C1180

© 2000 Cancer Research Campaign

Kennedy AS, Raleigh JA, Perez GM, Calkins DP, Thrall DE, Novotny DB and Varia MA (1997) Proliferation and hypoxia in human squamous cell carcinoma of the cervix: first report of combined immunohistochemical assays. *Int Radiat Oncol Biol Phys* **37**: 897–905

Koch CJ, Howell RL and Biaglow JE (1979) Ascorbate anion potentiates the cytotoxicity of nitro-aromatic compounds under hypoxic and anoxic conditions. *Br J Cancer* **39**: 321–329

Koch CJ, Evans SM and Lord EM (1995) Oxygen dependence of cellular uptake of EF5 [2-(2-nitro-1H-imidazol-1-y1)-N-(2,2,3,3,3-pentafluoropropyl) acetamide]: analysis of drug adducts by fluorescent antibodies versus bound radioactivity. *Br J Cancer* **72**: 869–874

Leith JT and Michelson S (1995) Secretion rates and levels of vascular endothelial growth factor in clone A or HCT-8 human colon tumor cells as a function of oxygen concentration. *Cell Prolif* **28**: 415–430

Levy AP, Levy NS, Loscalzo J, Calderone A, Takahashi N, Yeo KT, Koren G, Colucci WS and Goldberg MA (1995*a*) Regulation of vascular endothelial growth factor in cardiac myocytes. *Circ Res* **76**: 758–766

Levy AP, Levy Ns, Wegner S and Goldberg MA (1995*b*) Transcriptional regulation of the rat vascular endothelial growth factor gene by hypoxia. *J Biol Chem* **270**: 13333–13340

Levy AP, Levy NS and Goldberg MA (1996) Hypoxia-inducible protein binding to vascular endothelial growth factor mRNA and its modulation by the von Hippel–Lindau protein. *J Biol Chem* **271**: 25492–25497

Levy AP, Levy NS, Iliopoulos O, Jiang C, Kaelin WG Jr and Goldberg MA (1997) Regulation of vascular endothelial growth factor by hypoxia and its modulation by the von Hippel–Lindau tumor suppressor gene. *Kidney Int* **51**: 575–578

Li J, Perrella MA, Tsai JC, Yet S, Hsieh CM, Yoshizumi M, Patterson C, Endege WO, Zhou F and Lee ME (1995) Induction of vascular endothelial growth factor gene expression by interleukin-1 beta in rat aortic smooth muscle cells. *J Biol Chem* **270**: 308–312

Liu B, Earl HM, Baban D, Shoaibi M, Fabra A, Kerr DJ and Seymour LW (1995) Melanoma lines express VEGF receptor KDR and respond to exogenously added VEGF. *Biochem Biophys Res Commun* **217**: 721–727

Olive PL, Horsman M, Grau C and Overgaard J (1997) Detection of hypoxic cells in a $C_3H$ mouse mammary carcinoma using the comet assay. *Br J Cancer* **76**: 694–699

Olive PL, Johnston PJ, Banath JP and Durand RE (1998) The comet assay: a new method to examine heterogeneity associated with solid tumors. *Nat Med* **4**: 103–105

Parliament MB, Franko AJ, Allalunis-Turner MJ, Mielke BW, Santos CL, Wolokoff BG and Mercer JR (1997) Anomalous patterns of nitroimidazole binding adjacent to necrosis in human glioma xenografts: possible role of decreased oxygen consumption. *Br J Cancer* **75**: 311–318

Plate KH, Breier G, Weich HA and Risau W (1992) Vascular endothelial growth factor is a potential tumour angiogenesis factor in human gliomas in vivo. *Nature* **359**: 845–848

Plate KH, Breier G, Weich HA, Mennel HD and Risau W (1994) Vascular endothelial growth factor and glioma angiogenesis: coordinate induction of VEGF receptors, distribution of VEGF protein and possible in vivo regulatory mechanisms. *Int J Cancer* **59**: 520–529

Raleigh JA, Calkins-Adams DP, Rinker LH, Ballenger CA, Weissler MC, Fowler WC Jr, Novotny DB and Varia M (1998) Hypoxia and vascular endothelial growth factor expression in human squamous cell carcinomas using pimonidazole as a hypoxia marker. *Cancer Res* **58**: 3765–3768

Ryuto M, Ono M, Izumi H, Yoshida S, Weich HA, Kohno K and Kuwano M (1996) Induction of vascular endothelial growth factor by tumor necrosis factor $\alpha$ in human glioma cells. *J Biol Chem* **271**: 28220–28228

Shima DT, Deutsch U, and D'Amore PA (1995) Hypoxic induction of vascular endothelial growth factor (VEGF) in human epithelial cells is mediated by increases in mRNA stability. *FEBS Lett* **270**: 203–208

Shweiki D, Itin A, Soffer D and Keshet E (1992) Vascular endothelial growth factor induced by hypoxia may mediate hypoxia-initiated angiogenesis. *Nature* **359**: 843–845

Stein I, Neeman M, Shweiki D, Itin A and Keshet E (1995) Stabilization of vascular endothelial growth factor mRNA by hypoxia and hypoglycemia and coregulation with other ischemia-induced genes. *Mol Cell Biol* **15**: 5363–5368

Urtasun RC, Koch CJ, Franko AJ, Raleigh JA and Chapman JD (1986) A novel technique for measuring human tissue $pO_2$ at the cellular level. *Br J Cancer* **54**: 453–457

Vaisman N, Gospodarowicz D and Neufeld G (1990) Characterization of the receptors for vascular endothelial growth factor. *J Biol Chem* **265**: 19461–19466

Waleh NS, Brody MD, Knapp MA, Mendonca HL, Lord EM, Koch CJ, Laderoute KR and Sutherland RM (1995) Mapping of the vascular endothelial growth factor-producing hypoxic cells in multicellular tumor spheroids using a hypoxia-specific marker. *Cancer Res* **55**: 6222–6226

Wang GL, Jiang B-H, Rue EA and Semenza GL (1995) Hypoxia-inducible factor 1 is a basic-helix-loop-helix-PAS heterodimer regulated by cellular $O_2$ tension. *Proc Natl Acad Sci USA* **92**: 5510–5514

White FC, Carroll SM and Kamps MP (1995) VEGF mRNA is reversibly stabilized by hypoxia and persistently stabilized in VEGF-overexpressing human tumor cell lines. *Growth Factors* **12**: 289–301

Wizigmann-Voos S, Breier G, Risau W and Plate KH (1995) Up-regulation of vascular endothelial growth factor and its receptors in von Hippel–Lindau disease associated and sporadic hemangioblastomas. *Cancer Res* **55**: 1358–1364

*© 2000 Cancer Research Campaign*
